# Standardized Urine-Based Tuberculosis (TB) Screening With TB-Lipoarabinomannan and Xpert MTB/RIF Ultra in Ugandan Adults With Advanced Human Immunodeficiency Virus Disease and Suspected Meningitis

**DOI:** 10.1093/ofid/ofaa100

**Published:** 2020-03-24

**Authors:** Fiona V Cresswell, Jayne Ellis, Enock Kagimu, Ananta S Bangdiwala, Michael Okirwoth, Gerald Mugumya, Morris Rutakingirwa, John Kasibante, Carson M Quinn, Kenneth Ssebambulidde, Joshua Rhein, Edwin Nuwagira, Lillian Tugume, Emily Martyn, Caleb P Skipper, Conrad Muzoora, Daniel Grint, David B Meya, Nathan C Bahr, Alison M Elliott, David R Boulware

**Affiliations:** 1 Infectious Diseases Institute, Makerere University, Kampala, Uganda; 2 Clinical Research Department, London School of Hygiene and Tropical Medicine, London, United Kingdom; 3 MRC-UVRI-London School of Hygiene and Tropical Medicine Uganda Research Unit, Entebbe, Uganda; 4 Hospital for Tropical Diseases, University College London Hospitals NHS Foundation Trust, London, United Kingdom; 5 Division of Biostatistics, School of Public Health, University of Minnesota, Minneapolis, Minnesota, USA; 6 Microbiology Laboratory, Kiruddu Referral Hospital, Kampala, Uganda; 7 Division of Infectious Diseases and International Medicine, Department of Medicine, University of Minnesota, Minneapolis, Minnesota, USA; 8 Division of Infectious Diseases, Department of Medicine, University of Kansas, Kansas City, Kansas, USA; 9 Mbarara University of Science and Technology, Mbarara, Uganda; 10 Tropical Epidemiology Group, London School of Hygiene and Tropical Medicine, London, United Kingdom

**Keywords:** HIV, meningitis, TB-LAM, tuberculosis, Xpert MTB/RIF Ultra

## Abstract

**Background:**

Diagnosis of extrapulmonary tuberculosis (TB) remains challenging. We sought to determine the prevalence of disseminated TB by testing urine with TB-lipoarabinomannan (TB-LAM) lateral flow assay and Xpert MTB/RIF Ultra (Ultra) in hospitalized adults.

**Methods:**

We prospectively enrolled human immunodeficiency virus (HIV)-positive adults with suspected meningitis in Uganda during 2018–2020. Participants underwent standardized urine-based TB screening. Urine (60 mcL) was tested with TB-LAM (Alere), and remaining urine was centrifuged with the cell pellet resuspended in 2 mL of urine for Xpert Ultra testing.

**Results:**

We enrolled 348 HIV-positive inpatients with median CD4 of 37 cells/mcL (interquartile range, 13–102 cells/mcL). Overall, 26% (90 of 348; 95% confidence interval [CI], 21%–30%) had evidence of disseminated TB by either urine assay. Of 243 participants with both urine TB-LAM and Ultra results, 20% (48 of 243) were TB-LAM-positive, 12% (29 of 243) were Ultra-positive, and 6% (14 of 243) were positive by both assays. In definite and probable TB meningitis, 37% (14 of 38) were TB-LAM-positive and 41% (15 of 37) were Ultra-positive. In cryptococcal meningitis, 22% (40 of 183) were TB-LAM-positive and 4.4% (6 of 135) were Ultra-positive. Mortality trended higher in those with evidence of disseminated TB by either assay (odds ratio = 1.44; 95% CI, 0.83–2.49; *P* = .19) and was 6-fold higher in those with definite TB meningitis who were urine Ultra-positive (odds ratio = 5.67; 95% CI, 1.13–28.5; *P* = .04).

**Conclusions:**

In hospitalized Ugandans with advanced HIV disease and suspected meningitis, systematic screening with urine TB-LAM and Ultra found a high prevalence of urine TB test positivity (26%). In those with TB meningitis, urine tests were positive in over one third. There was little concordance between Ultra and TB-LAM, which warrants further investigation.

Tuberculosis (TB) is a major cause of death in people with human immunodeficiency virus (HIV). Human immunodeficiency virus-associated TB is often disseminated, defined as having 2 or more noncontiguous sites resulting from lymphohematogenous dissemination of *Mycobacterium tuberculosis* [1, 2]. Postmortem studies have shown that >90% of cases involve multiple organ systems including spleen, liver, multiple lymph node groups, kidneys, or central nervous system [3]. Diagnosis of disseminated TB is taxing due to challenges in accessing clinical samples from the affected sites. In addition, in the context of HIV-associated meningitis, of which cryptococcal meningitis is the most common cause [4, 5], disseminated TB may not be considered due to the considerable overlap in the clinical presentation of the 2 diseases: ie, fevers, night sweats, cough, and wasting. There are case reports and case series of cryptococcal meningitis coinfection with TB [6, 7], including TB meningitis [8], but no published studies systematically screened for TB among HIV-positive adults presenting with neurological infections. It is unknown to what extent undiagnosed disseminated TB infection contributes to the high mortality in HIV-infected patients with neurological infections.

Urine-based TB diagnostics have the potential to reduce missed TB diagnoses and reduce mortality in patients with advanced HIV disease. Urine is easily obtainable and can be tested using either a lateral flow assay (LFA) for TB-lipoarabinomannan (TB-LAM) or the Xpert MTB/RIF assay (Xpert; Cepheid, Sunnyvale, CA). Tuberculosis-LAM is emerging as the key urine-based TB diagnostic. Published data, including a 2016 Cochrane review, demonstrate that the first-generation Alere Determine TB-LAM Ag assay (TB-LAM; Alere, Waltham, MA) has a sensitivity of 40%–70% and a specificity of ≥98% in TB/HIV-coinfected patients with CD4 counts <100 cells/μL [9–11]. The STAMP trial evaluated the use of the Alere Determine TB-LAM Ag assay and Xpert MTB/RIF among HIV-positive patients hospitalized in Malawi and South Africa [12] and demonstrated reduced all-cause 56-day mortality with urine-based TB-LAM screening in certain high-risk subgroups of those with CD4 T-cell counts <100 cells/μL, severe anemia (hemoglobin <8 g/dL), or clinically suspected TB. These findings corroborate those of an earlier study that found a 4% (95% confidence interval [CI], 1%–7%) reduction in 8-week all-cause mortality in hospitalized HIV-positive adults with urine TB-LAM screening [13]. STAMP found that TB-LAM provided a 2-fold higher diagnostic yield than urine Xpert MTB/RIF. As a result, the World Health Organization (WHO) has recently updated their guidelines to support the expanded use of the Alere TB-LAM assay in all patients hospitalized with symptoms and signs of TB, CD4 count <200 cells/μL (irrespective of symptoms), or clinically advanced HIV disease, or who are seriously ill [14] .

Due to the limited sensitivity of urine Xpert MTB/RIF, urine screening with Xpert is not currently recommended by the WHO. In 2017, however, Cepheid introduced the re-engineered Xpert MTB/RIF Ultra (Ultra) with technical enhancements resulting in 7-fold improved analytical sensitivity and addition of a “trace” category for the lowest bacillary load [15]. Two diagnostic accuracy studies of Ultra on cerebrospinal fluid (CSF) in HIV-positive Ugandan adults with suspected TB meningitis found Ultra to be significantly more sensitive than Xpert MTB/RIF against a composite reference standard [16, 17]. However, publications on Xpert Ultra use in urine have thus far been limited, and include a case report [18] and a small number of samples in diagnostic accuracy studies of Ultra in extrapulmonary samples [19, 20], without evaluation as a screening tool. In a Ugandan study, the performance of urine Xpert Ultra in diagnosing pulmonary TB was assessed, and researchers found that Xpert Ultra had significantly higher sensitivity than TB-LAM, with 50% sensitivity in patients with CD4 <100 cells/µL [21]. This study, which also included HIV-negative patients, used sputum-based diagnostics as the reference standard. The suboptimal sensitivity of urine diagnostics in pulmonary TB diagnosis is perhaps not surprising because sputum studies are often discordant with urine diagnostics if the TB has not disseminated beyond the lungs, as may often be the case in HIV-negative patients [22]. In our study, we sought to determine the prevalence of disseminated TB infection by testing urine with the Alere TB-LAM LFA and Xpert Ultra in HIV-positive adults presenting with suspected meningitis.

## METHODS

### Study Population

We conducted a prospective cohort study as part of screening for an observational meningitis diagnostic study and a TB meningitis randomized controlled trial (RifT trial: ISRCTN 15668391). Consecutive HIV-infected adults presenting with suspected meningitis to Mulago National Referral Hospital, Kampala or Mbarara Regional Referral Hospital were screened for infectious meningitis etiologies using a stepwise diagnostic algorithm, as described elsewhere [23], after provision of written informed consent. Inclusion criteria for diagnostic testing were HIV-positive adults (≥18 years) presenting with suspected meningitis (eg, headache or altered mental status, clinical signs of meningism). From March 27, 2018 to February 6, 2020, urine-based screening for TB was conducted using an Alere Determine TB-LAM LFA assay (TB-LAM; Alere) and Xpert Ultra (Cepheid). Urine TB testing was conducted for all patients within 48 hours of admission to hospital. Tuberculosis screening was performed irrespective of TB symptoms, past or current TB therapy, or CD4 T-cell count among consented participants. Cryptococcal meningitis was diagnosed by positive CSF cryptococcal antigen (CrAg) LFA at the bedside and later confirmed by culture. Participants with suspected or confirmed TB meningitis were categorized according to the 2010 uniform case definition; with “definite TB meningitis” comprising those with microbiologically confirmed *M tuberculosis* in CSF and “probable TB meningitis” comprising those who score ≥10 points (or ≥12 points if brain imaging is performed) on clinical, CSF, and imaging criteria but who are negative for TB on CSF testing [24]. Due to its low specificity in a high HIV-burden setting “possible TB meningitis” was not included as a discrete diagnostic category.

### Study Procedures

A suggested minimum of 30 mL of midstream, early morning urine was collected from participants able to provide a sample. In the laboratory, 60 μL of unconcentrated urine was tested with the TB-LAM according to the manufacturers’ instructions, and test samples were classified according to the reference card with grade 1 or above considered positive according to the WHO guidelines [14]. The remainder of the urine specimen was measured using calibrated Falcon tubes, the volume was recorded, and the urine was processed for Ultra testing. The urine was concentrated by centrifugation at 3000 ×*g* for 15 minutes at room temperature. Surplus urine was decanted off leaving 2 mL in which the cell pellet was resuspended and loaded into the cartridge for Xpert Ultra testing. No buffer was used. Tuberculosis-LAM and Xpert Ultra testing were performed simultaneously by a laboratory technologist blinded to the patient history, and the TB-LAM result was reported independently without awareness of the Ultra result. Both results were reported in real-time to the clinical team.

Clinical management, including timing of commencement of anti-TB therapy and additional TB investigation, was at the discretion of the attending physician; wherever possible, anti-TB therapy was started as soon as possible for all patients with a positive TB-LAM and/or Xpert Ultra result, and additional work-up, including collection of additional clinical samples where possible for TB microbiology (Xpert Ultra and/or Mycobacteria Growth Inhibitor Tube [MGIT] culture; Becton Dickinson, Franklin Lakes, NJ), chest radiography, and abdominal ultrasound, was performed where indicated and feasible.

Baseline demographics, clinical characteristics including antiretroviral therapy (ART) use, and laboratory data were collected as part of the screening process. Consented participants were followed up until hospital discharge or through >10 weeks if enrolled into a meningitis clinical trial.

### Ethics

All participants (or a surrogate in cases of mental incapacity) provided written informed consent for urine testing, lumbar puncture, CSF testing, CSF storage, and data collection. Approval for the studies was obtained from the Mulago Hospital Research Ethics Committee (MHREC1260 and MHREC1246), Uganda National Council for Science and Technology, University of Minnesota, and the London School of Hygiene and Tropical Medicine.

### Statistical Analysis

Statistical analysis is primarily descriptive with the primary endpoint of interest being the prevalence of disseminated TB detected by urine testing. We compared baseline characteristics across meningitis etiologies, with Kruskall-Wallis tests comparing continuous variables and χ ^2^ tests comparing categorical variables. We compared the proportion of positive Urine TB-LAM results and the distribution of TB-LAM grade across meningitis etiologies using a χ ^2^ test. We compared congruency in positive TB-LAM versus Xpert Ultra tests by McNemar’s test, and we compared the distribution of urine Xpert Ultra semiquantitative grade across meningitis etiologies using a χ ^2^ test and Fisher’s exact test (as appropriate according to sample size). We tested the relationship between urine volume and a positive Xpert Ultra result using a χ ^2^ test with volume dichotomized at the median 24 mL (range, 4.5–45 mL). We reported the proportion surviving hospitalization by each urine TB diagnostics result, and we compared test results and mortality using univariable logistic regression. *P* values were not adjusted for multiple testing. All analyses were conducted using SAS (version 9.4; SAS Institute, Cary, NC).

## RESULTS

### Patient Demographics

We enrolled 348 HIV-positive hospitalized participants, 58% (201 of 348) of whom were men. The median age was 35 years (interquartile range [IQR], 29–42). The study participants had advanced HIV disease (median CD4 = 37 cells/μL; IQR, 13–102 cells/mcL), despite 48% (167 of 347) reporting ART use at the time of presentation (implying virologic failure, suboptimal adherence, or recent initiation). The majority of participants had cryptococcal meningitis (57%, 198 of 348); 18% (62 of 348) had definite or probable TB meningitis according to the uniform clinical case definition [24]. Cerebrospinal fluid features by meningitis etiology are shown in Table 1.

**Table 1.** Participant Baseline Demographics, Clinical, and CSF Findings by Disease Category

Characteristics	Cryptococcal Meningitis	TBM (Confirmed)	TBM (Probable)	Unknown Other^a^
N	198	40	22	88
Age, years	35 [29–42]	32 [28–36]	38 [30–48]	37 [30–44]
Women	79 (40%)	17 (43%)	11 (50%)	40 (45%)
HIV Factors				
CD4^+^ T cells/μL	23 [8–70]	72 [41–111]	55 [22–186]	205 [45–340]
Currently on ART	91 (46%)	17 (43%)	8 (38%)	51 (58%)
Months on ART^b^	2.3 [0.6–29.4]	2.2 [0.7–27.9]	1.5 [0.5–11.3]	6.9 [1.6–73.9]
Clinical				
Fever	77 (52%)	34 (97%)	20 (91%)	46 (68%)
Headache	190 (98%)	34 (94%)	15 (68%)	61 (76%)
Duration of headache	14 [7–21]	14 [8–21]	14 [7–21]	14 [7–30]
Focal neurologic deficit	7 (4%)	15 (38%)	8 (36%)	21 (24%)
Wasting	81 (42%)	23 (58%)	13 (59%)	35 (41%)
Glasgow come score <15	70 (35%)	36 (92%)	19 (86%)	45 (52%)
Seizure	41 (30%)	11 (38%)	4 (21%)	23 (38%)
Weight, kg	52 [45–60]	55 [45–58]	53 [50–60]	50 [50–59]
Cough	56 (39%)	25 (74%)	10 (53%)	39 (61%)
Night sweats^c^	31 (25%)	22 (85%)	13 (72%)	24 (44%)
Baseline CSF Results				
Opening pressure, cm H_2_O	23 [14–32]	19 [10–29]	15 [10–24]	18 [12–23]
Opening pressure <20 cm H_2_O	66 (38%)	16 (52%)	10 (67%)	40 (62%)
Total WBC count cells μL	<5 [<5–50]	30 [<5–220]	<5 [<5–200]	<5 [<5–30]
Total WBC count <5 μL	117 (60%)	17 (45%)	11 (52%)	61 (71%)
CSF protein, mg/dL	69 [36–100]	128 [61–200]	88 [31–151]	68 [28–111]
CSF WBC <5 μL and protein <45 mg/dL	51 (28%)	5 (15%)	4 (20%)	26 (36%)

Abbreviations: ART, antiretroviral therapy; CSF, cerebrospinal fluid; HIV, human immunodeficiency virus; IQR, interquartile range; TBM, tuberculous meningitis; WBC, white blood cells.

NOTE: Data are n (%) or median [IQR].

^a^Includes possible and nontuberculous meningitis diagnoses.

^b^Among those on ART at diagnosis.

^c^Data on night sweats were only available for 122 of cryptococcosis, 26 definite TB meningitis, 18 probable TB meningitis, and 54 other.

### Diagnostic Yield of Urine Tuberculosis-Lipoarabinomannan and Ultra

Overall, 26% (90 of 348; 95% CI, 21%−30%) of the study participants had evidence of disseminated TB by positive TB urine assay: 23% (75 of 327; 95% CI, 18%−27%) positive by urine TB-LAM, and 11% (29 of 264; 95% CI, 7%−15%) positive by urine Xpert Ultra. In cryptococcal patients, 22% (40 of 183) had evidence of disseminated TB by urine TB-LAM and 4.4% (6 of 135) by urine Xpert Ultra. In definite TB meningitis patients, 37% (14 of 38) had a positive urine TB-LAM and 41% (15 of 37) had a positive urine Xpert Ultra. In probable TB meningitis patients, 41% (9 of 22) had a positive urine TB-LAM and 21% (4 of 19) had a positive on urine Xpert Ultra. In a composite of definite and probable TB meningitis, 37% (23 of 62) had a positive urine TB-LAM and 34% (19 of 56) had a positive Xpert Ultra (Table 2 and Supplemental Figure 1). Among urine TB-LAM-negative subjects, the number needed to test with Xpert Ultra to diagnose an additional case of disseminated TB was 13 persons (95% CI, 8.0−22.9).

**Table 2.** Urine TB Diagnostic Test Results by Disease Category

Urine TB Testing Details	Cryptococcal Meningitis	TBM (Confirmed)	TBM (Probable)	Unknown Other^a^	*P* Value^b^
N	198	40	22	88	
Urine TB-LAM					
Tested with TB-LAM, N	183	38	22	84	
Urine TB-LAM positive, n (%)	40 (21.9%)	14 (36.8%)	9 (40.9%)	12 (14.3%)	<.01
LAM Grade 1	25 (13.7%)	5 (13.2%)	4 (18.2%)	7 (8.3%)	
LAM Grade 2	9 (4.9%)	0 (0.0%)	4 (18.2%)	4 (4.8%)	
LAM Grade 3	5 (2.7%)	7 (18.4%)	1 (4.5%)	0 (0.0%)	
LAM Grade 4	1 (0.5%)	2 (5.3%)	0 (0.0%)	1 (1.2%)	
Urine Xpert Ultra					
Tested with Xpert Ultra, N	135	37	19	73	
Urine Xpert Ultra positive, n (%)	6 (4.4%)	15 (40.5%)	4 (21.1%)	4 (5.5%)	<.001
Semiquantitative trace	1 (0.7%)	4 (10.8%)	1 (5.3%)	0 (0.0%)	
Semiquantitative very low	1 (0.7%)	4 (10.8%)	0 (0.0%)	1 (1.4%)	
Semiquantitative low	2 (1.5%)	4 (10.8%)	3 (15.8%)	0 (0.0%)	
Semiquantitative medium	0 (0.0%)	2 (5.4%)	0 (0.0%)	1 (1.4%)	
Grade unknown	2 (1.5%)	1 (2.7%)	0 (0.0%)	2 (2.7%)	
Either TB Test Positive					
TB-LAM and/or Xpert Ultra positive	44 (22%)	20 (50%)	11 (50%)	15 (17%)	<.001

Abbreviations: TB, tuberculosis; TB-LAM, TB-lipoarabinomannan; TBM, tuberculous meningitis.

^a^Includes possible and nontuberculous meningitis diagnoses.

^b^ *P* value assessed by χ ^2^ distribution of LAM positivity and Ultra positivity across disease groups.

### Concordance Between Tuberculosis-Lipoarabinomannan and Ultra

**Figure 1.**
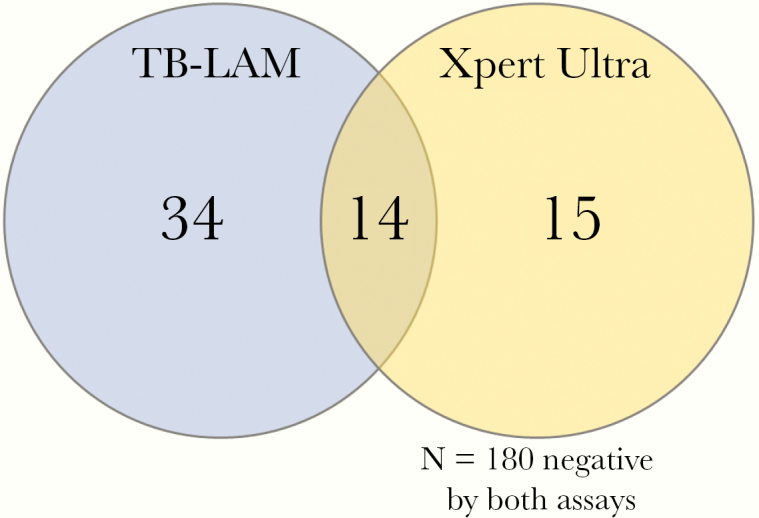
Venn diagram of overlap between tuberculosis (TB)-positive urinary assays. Of 243 persons with both TB-lipoarabinomannan (TB-LAM) and urine Xpert Ultra results, 63 were positive by either TB urinary assay, 76% (48 of 63) of whom were positive by TB-LAM and 46% (29 of 63) by Xpert Ultra (*P* = .01). Only 22% (14 of 63) of those with a positive urine TB test were positive by both TB-LAM and Ultra. Among the 195 persons with a negative TB-LAM, 15 (7.7%) were positive by Xpert Ultra, with a number needed to test with Ultra to diagnose an additional case of disseminated TB being 13 (95% CI, 8.0–22.9).

**Figure 2.**
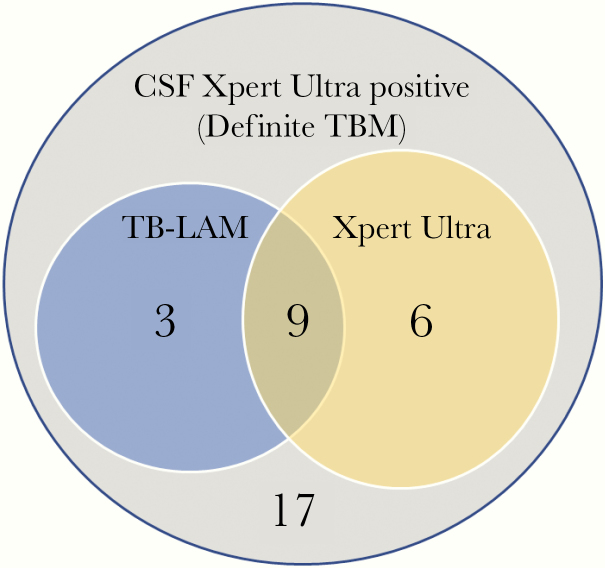
Venn diagram illustrating overlap of tuberculosis-lipoarabinomannan (TB-LAM) and urine Ultra among those with definite TB meningitis (TBM). Of 35 persons with definite TBM tested with both assays, a total of 18 (51%) had positive urine test, 12 (34%) were urine TB-LAM positive, and 15 (43%) were urine Ultra positive with an overlap of 9 of 18 (50%) positive participants being positive by both assays. Overall, 49% (17 of 35) with definite TBM, diagnosed by positive CSF Xpert Ultra, had negative urinary tests.

Overall, 243 participants had both urine TB-LAM and Xpert Ultra results available for paired analysis. Of 63 participants with a positive urine TB assay, 76% (48 of 63) were positive by TB-LAM and 46% (29 of 63) were positive by Xpert Ultra (*P* = .01). Only 22% (14 of 63) of those with a positive urine TB test were positive by both TB-LAM and Ultra (Figure 1). The overlap of TB-LAM and Ultra was greater among those with definite TB meningitis. Of the 35 definite TB meningitis participants tested with both assays, 51% (18 of 35) had positive urine test, 34% (12 of 35) were urine TB-LAM positive, and 43% (15 of 35) were urine Ultra positive with an overlap of 50% (9 of 18 of positive tests). Overall, 17 persons with microbiologically confirmed TB meningitis had negative urinary tests (Figure 2).

### Urine Diagnostics as a Predictor of In-Hospital Mortality

Among those who are known to have died in hospital, the median time to death was 7 days (IQR, 3–12 days). Among participants with disseminated TB with either urine assay positive, the mortality was 33% (27 of 82), compared with 25% (59 of 232) among those with negative urine assays (odds ratio = 1.44; 95% CI, 0.83–2.49; *P* = .19). The mortality was also higher (albeit nonsignificantly) among participants with positive urine TB-LAM (32% positive LAM vs 26% negative LAM, *P* = .32) and among urine Xpert Ultra positive participants (36% vs 24%, *P* = .18). However, among those with definite TB meningitis, there was 50% mortality (7 of 14) in those with positive urine Xpert Ultra compared with 23% (3 of 20) in those with negative urine Xpert Ultra (odds ratio = 5.67; 95% CI, 1.13–28.5; *P* = .04). With either urine TB-LAM or Ultra positive, the odds of dying were 3-fold higher (odds ratio = 3.00; 95% CI, 0.71–12.7; *P* = .14) in those with definite TB meningitis (Table 3).

**Table 3.** In-Hospital Mortality by Urine Diagnostic Result and Meningitis Aetiology by Univariate Logistic Regression^a^

	Alere TB-LAM				Xpert MTB/RIF Ultra				Either TB Assay Positive^b^	
Study Population	Positive	Negative	Odds Ratio (95% CI)	*P* Value	Positive	Negative	Odds Ratio (95% CI)	*P* Value	Odds Ratio (95% CI)	*P* Value
Overall	32% (22 of 68)	26% (60 of 229)	1.35 (0.75–2.42)	.32	36% (10 of 28)	24% (51 of 214)	1.78 (0.77–4.09)	.18	1.44 (0.83–2.49)	.19
Cryptococcal	32% (12 of 38)	27% (36 of 133)	1.24 (0.57–2.72)	.59	50% (3 of 6)	23% (28 of 121)	3.32 (0.63–17.4)	.16	1.41 (0.67–2.96)	.37
TBM, definite	38% (5 of 13)	32% (7 of 22)	1.34 (0.32–5.61)	.69	50% (7 of 14)	15% (3 of 20)	5.67 (1.13–28.5)	.04	3.00 (0.71–12.7)	.14
TBM, probable	50% (4 of 8)	38% (5 of 13)	1.60 (0.27–9.49)	.60	0% (0 of 4)	50.0% (7 of 14)				
Other/unknown	11.1% (1 of 9)	19.7% (12 of 61)	0.51 (0.06–4.48)	.54	0% (0/4)	22% (13/59)			0.34 (0.04–2.84)	.32

Abbreviations: CI, confidence interval; TB, tuberculosis; TB-LAM, TB-lipoarabinomannan; TBM, tuberculous meningitis.

^a^Data represent mortality by TB test status. Hospital outcome was unknown for 34 participants (7.5% of cryptococcal, 7.5% TBM definite, 5% TBM probable, 17% unknown/other meningitis). Among those who are known to have died in hospital, the median time to death was 7 days (interquartile range, 3–12 days).

^b^Either TB assay positive is any positive urine diagnostic (TB-LAM or Ultra or both) versus both assays being negative.

### Relationship Between Urine Volume and Ultra Positivity

Among patients with urine volume measured and recorded, 13% (8 of 64) of patients with <24 mL tested positive by Ultra, and 9% (6 of 65) of patients with ≥24 mL tested positive by Ultra (*P* = .55). The distribution of urine volume among those with definite TB meningitis was the same as the rest of the study population, thus larger centrifuged volumes did not yield a higher prevalence of detecting TB.

We tested for differences in the demographics, clinical, or laboratory parameters of patients who were positive by a single urine assay or both assays. We explored available laboratory parameters by urine test positivity, and there were no significant differences in baseline CD4 count, hemoglobin, or creatinine level in patients who had discordant results (Supplementary Table 1).

## DISCUSSION

We have demonstrated that the prevalence of urine TB test positivity is 26% amongst HIV-infected patients presenting to hospital with suspected meningitis and advanced HIV disease in Uganda. The urine TB-LAM assay had more positive results (23%) than urine Xpert Ultra (11%), yet Xpert Ultra positivity was associated with higher risk of mortality (36% in-hospital mortality), particularly in those with TB meningitis (50% in-hospital mortality). Uganda has a high TB incidence rate (200 of 100 000 population in 2018); our findings may be generalizable to many other countries in sub-Saharan Africa with similar TB incidence rates [25].

Cryptococcosis and TB coinfection (including TB meningitis) has been described in case reports, and in the Cryptococcal Optimal ART Timing (COAT) trial where 18% (32 of 177) of participants with cryptococcal meningitis were treated for concurrent active TB disease during the 46-week study period [26]. However, this is the first study in which disseminated TB has been systematically screened for in an HIV-associated meningitis cohort. Among the 198 cryptococcal meningitis patients, one quarter had a positive urine TB test, TB-LAM (22%) or Xpert Ultra (4.4%). The true prevalence of disseminated TB could be higher than this given that urine TB testing may not detect all forms of disseminated TB. Given the considerable overlap in symptoms between cryptococcal meningitis and disseminated TB in advanced HIV disease, it is likely that without routine screening for TB, some of these diagnoses would be missed, and therefore they may contribute to the high mortality associated with cryptococcal meningitis. Consistent with other studies, in-hospital mortality was high in our cryptococcal meningitis subgroup and trended towards there being persistent increased mortality in coprevalent disseminated TB infection despite clinical teams having real-time access to TB urine diagnostic results; however, this study was not designed nor powered to determine an impact of coprevalent TB on mortality.

We investigated the clinical utility of TB urine diagnostics in TB meningitis, a condition that is notoriously challenging to diagnose even with molecular CSF diagnostics. Overall, 50% (20 of 40; with both urine tests) of definite TB meningitis participants had either a positive TB-LAM or urine Xpert Ultra. Among probable TB meningitis cases, patients with highly suggestive clinical features but in whom rapid CSF diagnostics (acid-fast bacilli [AFB] smear and CSF Xpert Ultra) were negative, 41% were urine TB-LAM positive and 21% urine Ultra positive. A lack of sensitive and timely diagnostics has historically made TB meningitis diagnoses extremely difficult, and therefore treatment for probable TB meningitis rests upon the presence of symptoms or signs indicative of meningitis, CSF features plus suggestive imaging criteria or confirmed evidence of extra-neural TB. The uniform case definition agreed by consensus in 2010 does not include the TB-LAM assay as “evidence of extra-neural TB” [24], but our data suggest that this may be warranted. In this study a positive urine TB test was interpreted as evidence of extra-neural TB when assigning points in the uniform case definition. Urine TB diagnostics have the potential to improve our classification of TB meningitis cases and warrant investigation in a larger study. Although not routinely used as part of a TB meningitis diagnostic work-up, urine is an easily obtained sample, and TB-LAM and Ultra are relatively low cost; therefore, we advocate their use as adjunctive tests in the investigation of patients with suspected TB meningitis.

Urine Xpert Ultra has previously been used to diagnose disseminated TB in a case report [18] and has been evaluated as a marker of pulmonary TB in a cross-sectional study [21], but this is the first time that urine Xpert Ultra has been evaluated as a screening test for disseminated TB in a prospective cohort and correlated with survival. Urine Xpert Ultra was positive in 11% of our (tested) cohort overall, demonstrating that urine is an additional viable clinical sample for use with Ultra. It is interesting to note that in patients with definite TB meningitis, Xpert Ultra and TB-LAM were positive in a similar proportion of patients (41% and 37% respectively), whilst there were considerably more positive TB-LAM tests than Xpert Ultra tests in patients with cryptococcal meningitis (22% and 4% respectively). Our data raise several questions about the MTB antigen load required for respective test positivity, the performance of urine Xpert Ultra at different degrees of immunosuppression, the impact of renal TB infection versus TB bacteremia on test performance, and the specificity of TB-LAM in patients with disseminated fungal diseases or other opportunistic infections [27].

In our subgroup analysis, in-hospital mortality was 6-fold higher (50% vs 15%; odds ratio = 5.67; 95% CI, 1.13–28.5; *P* = .04) in patients with definite TB meningitis and a positive urine Xpert Ultra in comparison to patients with definite TB meningitis and a negative urine tests. These data suggest that urine Ultra testing may be a useful test to identify TB meningitis patients with at particularly higher risk of death and thus requiring more intensive TB therapy or alternative or adjunctive host-directed immunotherapy. Future research is needed to explore whether urine Ultra positivity and the increased mortality relates to burden of MTB infection, host factors, or both.

Overall, there was little concordance between urine Xpert Ultra and TB-LAM positivity, only 22% of those positive by either test were positive by both assays. A similar observation was made in the STAMP trial, in which only 23% of patients had both urine TB-LAM and urine Xpert MTB/RIF positivity [12]. The reasons for this are unclear. We observed no statistically significant differences in the demographics, clinical, or laboratory parameters of patients who were positive by a single urine assay or both assays, although our study was not powered to detect a clear association between these variables and urine assay positivity. We need to further investigate the lack of concordance between TB-LAM and urine Xpert Ultra and to explore whether this relates to the following: (1) variation in the *M tuberculosis* burden required for test positivity; (2) interpatient differences in the renal parenchymal changes associated with renal TB or HIV-related nephropathy; or (3) differences in host immunity that may impact on the degree of immune complexing of circulating TB-LAM antigen and thereby its potential to cross the basement membrane [28]. Such a study could include immunological and renal parameters (such as proteinuria or urine-protein to creatinine ratio) to investigate whether the degree of immunodeficiency or glomerular pathology may contribute towards the likelihood of excreting *M tuberculosis* bacilli or the TB-LAM glycolipid—the latter is only 17.5 kDa without immune complexing but 150–1000 kDa when complexed with anti-LAM antibodies—into the urine.

One strength of our study is that we studied a large cohort of 348 adults and this is the first time that urine TB diagnostics have been evaluated in a prospective manner among HIV-associated meningitis patients. However, we do recognize limitations to our study. Due to either suboptimal volume of urine sample or due to Ultra test availability, not all patients had TB-LAM and Xpert Ultra test results available for analysis. In instances in which urine volume was limited, TB-LAM testing was prioritized as per WHO guidelines, and overall more TB-LAM tests were conducted because bedside TB-LAM tests were consistently more available than Ultra. To ensure a fair comparison of the diagnostic performance of TB-LAM and urine Xpert Ultra, the concordance and diagnostic yield of each test was only compared in 243 participants who had both test results available. Second, due to the high rates of urinary incontinence in this critically ill population, not all participants managed to provide the suggested >30 mL of urine. Low urine volume may have impacted the sensitivity of Ultra, although we did not detect a difference in TB test positivity in urine samples above and below the median 24-mL volume. Third, our study was exploratory and not powered to detect a clear association between TB-LAM and/or Xpert Ultra positivity and death. Although there was a trend towards worse outcomes in patients with evidence of disseminated TB, our data herein provide estimates necessary for future sample size calculations. Finally, due to the study setting and population (critically ill meningitis patients), we were unable to perform an extensive array of other TB diagnostics tests such as imaging or sputum collection to corroborate the urine results. However, urine TB-LAM has a high specificity (≥98% in TB/HIV-coinfected patients), and although there is a lack of published data on the specificity of urine Ultra, Ultra has a high specificity when used on other clinical samples [11, 15]; therefore, in this study population with advanced HIV disease in a setting known to have a high prevalence of TB, we believe the risk of false-positive results is minimal. The duration of urine Xpert Ultra positivity after prior TB treatment is unknown, but given the continuous filtration of the kidneys and that urine is normally a sterile fluid, it is unlikely that nonviable bacilli would persist months or years after prior treatment, although further study of this point is warranted.

## CONCLUSIONS

In conclusion, we have demonstrated a 26% prevalence of positive urine TB tests in patients presenting with HIV-associated meningitis. Our results support the expanded use of TB urine diagnostics in patients with meningitis to optimize timely diagnosis and treatment of TB, especially, but not only, in settings such as sub-Saharan Africa where TB infection prevalence is extremely high. Further studies are required to investigate whether disseminated TB is an independent risk factor for death in cryptococcal meningitis and whether enhanced TB treatment can improve outcomes. We have also shown that urine TB diagnostics are of use in TB meningitis; first as rapid, noninvasive adjunctive tests for suspected TB meningitis and second, possibly, as a prognostic tool in patients with HIV-associated TB meningitis. Further research is required to investigate the lack of concordance between urine TB-LAM and urine Xpert Ultra.

## Supplementary Data

Supplementary materials are available at *Open Forum Infectious Diseases* online. Consisting of data provided by the authors to benefit the reader, the posted materials are not copyedited and are the sole responsibility of the authors, so questions or comments should be addressed to the corresponding author.

**Supplementary Table 1.** Demographic, HIV, clinical, and laboratory factors by urine TB diagnostic result.

ofaa100_suppl_Supplementary_Figure_1

ofaa100_suppl_Supplementary_Table_1
