# Supplementary figures and images for: Standardized Urine-Based Tuberculosis (TB) Screening With TB-Lipoarabinomannan and Xpert MTB/RIF Ultra in Ugandan Adults With Advanced Human Immunodeficiency Virus Disease and Suspected Meningitis
Source: Open Forum Infect Dis. 2020 Mar 24;7(4):ofaa100. doi: 10.1093/ofid/ofaa100 (PMC7192026; doi:10.1093/ofid/ofaa100)

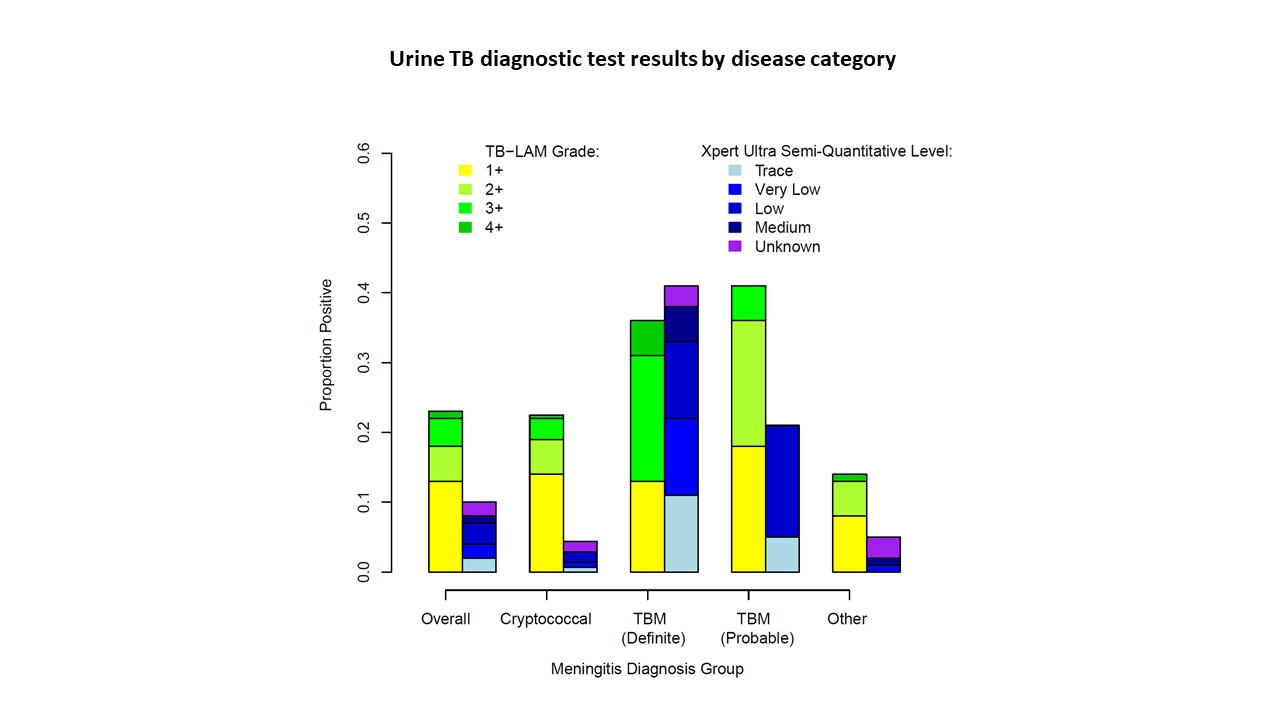

Supplement: ofaa100_suppl_Supplementary_Figure_1 [file ofaa100_suppl_supplementary_figure_1.png]
