# Supplementary material for: Standardized Urine-Based Tuberculosis (TB) Screening With TB-Lipoarabinomannan and Xpert MTB/RIF Ultra in Ugandan Adults With Advanced Human Immunodeficiency Virus Disease and Suspected Meningitis
Source: Open Forum Infect Dis. 2020 Mar 24;7(4):ofaa100. doi: 10.1093/ofid/ofaa100 (PMC7192026; doi:10.1093/ofid/ofaa100)
Supplement: ofaa100_suppl_Supplementary_Table_1 [file ofaa100_suppl_supplementary_table_1.pdf]

## Supplementary table 1. Demographic, HIV, clinical and laboratory factors by urine TB diagnostic result

| Characteristics | Positive by TB-LAM and Ultra N=14 | Positive TB-LAM only N=34 | Positive Ultra only N=15 | Both negative N=180 | p-value[1] |
|---|---|---|---|---|---|
| Age (years) | 36 [32, 41] | 33 [28, 38] | 37 [30, 42] | 35 [29, 40] | 0.40 |
| Female | 5 (35.7%) | 12 (35.3%) | 9 (60.0%) | 80 (44.4%) | 0.40 |
| **HIV factors** | | | | | |
| CD4+ cell count | 18 [8, 30] | 27 [9, 86] | 52 [45, 79] | 52 [15, 155] | 0.09 |
| Currently on ART | 4 (28.6%) | 13 (38.2%) | 8 (53.3%) | 99 (55.3%) | 0.09 |
| Months on ART[2] | 0.3 [0.2, 0.7] | 1.0 [0.5, 4.2] | 1.8 [0.6, 28.4] | 3.5 [1.0, 35.0] | 0.03 |
| Tenofovir at screening | 5 (100.0%) | 16 (94.1%) | 5 (71.4%) | 90 (81.1%) | 0.38 |
| **Clinical** | | | | | |
| Fever | 11 (91.7%) | 16 (64.0%) | 10 (83.3%) | 87 (62.1%) | 0.10 |
| Headache | 10 (83.3%) | 24 (77.4%) | 12 (85.7%) | 158 (91.3%) | 0.08 |
| Duration of headache | 10.5 [7.0, 14.0] | 14.0 [7.0, 30.0] | 14.0 [7.0, 20.5] | 14.0 [7.0, 21.0] | 0.11 |
| Focal neurologic deficit | 4 (28.6%) | 1 (2.9%) | 4 (26.7%) | 36 (20.0%) | 0.02 |
| Wasting | 10 (71.4%) | 11 (32.4%) | 8 (53.3%) | 66 (37.5%) | 0.05 |
| GCS < 15 | 8 (57.1%) | 17 (50.0%) | 10 (66.7%) | 88 (48.9%) | 0.58 |
| Seizure | 3 (27.3%) | 7 (29.2%) | 4 (44.4%) | 45 (36.9%) | 0.78 |
| Weight (kg) | 50.0 [47.0, 55.0] | 51.0 [50.0, 57.0] | 50.0 [50.0, 65.0] | 54.0 [48.0, 60.0] | 0.64 |
| Cough | 10 (71.4%) | 12 (44.4%) | 6 (66.7%) | 66 (50.4%) | 0.32 |
| Night sweats | 7 (70.0%) | 10 (47.6%) | 3 (42.9%) | 42 (38.5%) | 0.26 |
| **Laboratory parameters** | | | | | |
| CSF opening pressure (cm $H_2O$) | 14 [10, 23] | 20 [13, 25] | 14 [11, 36] | 20 [13, 29] | 0.28 |
| Total CSF WBC count (cells/μL) | 4.0 [4.0, 25.0] | 4.0 [4.0, 27.5] | 45.0 [4.0, 220.0] | 4.0 [4.0, 55.0] | 0.26 |
| Total CSF WBC count < 5 | 9 (64.3%) | 22 (68.8%) | 7 (46.7%) | 116 (66.3%) | 0.48 |
| CSF protein | 79 [25, 131] | 31 [23, 95] | 111 [48, 188] | 76 [35, 119] | 0.02 |
| Creatinine (mg) | 0.74 [0.52, 1.09] | 0.90 [0.67, 1.07] | 0.61 [0.55, 0.68] | 0.76 [0.54, 1.00] | 0.25 |
| Hemoglobin (g/dL) | 9.8 [7.8, 12.4] | 10.6 [8.4, 11.5] | 12.0 [10.4, 12.8] | 11.1 [9.1, 12.9] | 0.55 |

Data is presented as median [IQR] or N (%)

[1] Kruskal-Wallis test for medians; chi-square or exact test for proportions.

[2] Among those on ART at diagnosis
